# The Story of the Liver, by Dr. Andrew Wilson

**Published:** 1896-02

**Authors:** Arthur Fisher

**Affiliations:** M. D., L. R. C. S., Edin., Montreal, Canada


### "THE STORY OF THE LIVER, BY DR. ANDREW WILSON."

ARTHUR FISHER, M. D., L. R. C. S., EDIN., MONTREAL, CANADA.

I met with a popular article with this heading in *Harper's New Monthly Magazine* for May, 1895, page 957, the perusal of which I would earnestly recommend to your readers, as bearing wonderfully on Homœopathy. Although written for the laity, I am not ashamed to confess that, though a teacher of anatomy and physiolcgy (true, some fifty years ago), I found a vast deal in it not only to freshen my memory, but also to supply a knowledge in which I found myself deficient. No doubt Dr. Wilson has expressed his views in medical publications, but in *Harper's* they are easily obtainable by all.

He finishes his article thus: "The story of the liver, thus briefly narrated, forms, perhaps, one of the most typical illustrations of the extent and nature of the researches in which the science of these latter days is given to engage. It is often difficult for those whose interests lie outside the domain of scientific research to admit the utility of investigations, which, when casually viewed, appear to be far removed from any practical application to human affairs, but the house of knowledge is only builded by slow degrees and by many hands, and the liver's story finds its best commentary and moral in the fact that on the foundation afforded by such studies the edifice of rational medicine is reared, and this result in turn makes, directly for the cure of disease and for the promotion of the happiness of man."

We may take exception to the statement that the result makes directly for the cure of disease and for the promotion of the happiness of man. Most of your readers will hardly admit that much more of what the doctor is ignorant is not wanting for the cure of disease and the happiness of man.

The anatomy and physiology of the liver and digestive organs are amply and intelligently portrayed. Thus, he says regarding the liver cells themselves, they are, of course, utterly microscopic bodies. In diameter they vary from the one-thousandth part to the one two-thousandth part of an inch. Of yellow color, the microscope shows us that their protoplasm or living matter is of granular nature and exhibits oil globules in its substance.

If we have regard to the liver's size, these facts regarding its constitution become invested with singular interest, for the gland must be composed of millions of these living cells, or workmen, whose collective labors represent the actual work the liver performs.

Farther on he remarks, "Cells of one kind discharge one duty, be it respectively the formation of bone, the growth of muscle, or the secretion of gastric juice, and they discharge this duty only. It is left for cells of another kind to manufacture saliva, produce nerve force, or make tears."

Now, when we consider the infinite minuteness of the cells of which the whole of our bodies are composed, and remember that each cell is supplied with blood-vessels, nerves, and secreting tubes, can it any longer appear to us extraordinary that our medicines should need to be reduced to a division equally minute to enable them to permeate and act on the tissues? This, then, is the first lesson which the doctor unwittingly teaches us.

Passing over the doctor's admirable anatomical and physiological descriptions, we come to functions which more immediately concern us homœopaths. Here he says, " Perhaps the most natural method of appreciating the study of the liver is that of commencing our study with the relations of the gland to the digestion of our food. Here we fall back on an elementary piece of physiology which leads us straight to the nature of our foods, and to the work of the stomach in their assimilation. People are often startled to learn that the stomach has really very little to do, as regards the bulk of the operation, with the digestion of food at all. But such is the case. By far the greater part of every meal we eat consists of starches, sugars, and fats, and over these articles of diet, all important as they are as energy-producing foods, the stomach exercises little or no digestive power. There is, however, another class of foods of highly important kind which is the special care of the stomach. These are the nitrogenous foods which go to build up the tissues of our frame, whereof albumen represented by the piece of meat, and white of egg, the gluten of flour, and the casein of milk, and like substances, are familiar examples. It is the nitrogenous foods which the stomach, by aid of its gastric juice, poured out on the foods, is able chemically to alter and to adapt for their further assimilation. Let us see, then, what happens when the work of the stomach fails to be carried out. The nitrogenous foods mixed with the gastric juice (whereof the chief constituents are pepsin and hydrochloric acid) are converted into *peptones.*

This action is a complex one, suffice to say that at its close peptones are formed out of the albumens and like foods which the meal has contained. Now, what are these peptones? The answer is they are still albuminous bodies, chemically speaking, but changed physically in a marked degree from the state in which they were swallowed. For in the shape of peptones they can pass easily by diffusion through the walls of the stomach, and are thus taken up by the blood-vessels of that organ. So that we arrive at the real *raison d'etre* of the stomach. In a physiological sense, if we say that its chief duty is to convert our nitrogenous food into peptones so that in this form they may strain through the walls of the stomach and gain admittance to the blood, carrying our thoughts back to the arrangements of the liver, it will be remembered that the portal vein which enters the liver is made up of a union of veins coming from the stomach intestine and other digestive organs. Therefore it follows that the peptones of our food, passing from the stomach into its blood-vessels will be carried by these vessels into the portal vein, and will be swept up with the blood-current of that vessel into the liver. This in itself is an interesting point, for it teaches us in plain language that the stomach is a short cut for these nitrogenous foods into the blood, only the short cut leads through the liver, and here begins the recital of one of the chief duties of that gland. The stomach's office is by means of the gastric juice to arrest the nitrogenous foods, which it passes on as peptones into the depot (or liver) while the fats, starches, and sugars are allowed to go on into the intestines which is their own and proper digestive destination.

One of the most remarkable facts regarding the natural digestion of our food which modern science has disclosed is that which declares on experimental evidence that under certain conditions our diet may poison us. This strange but true declaration applies to these very peptones. The evidence on this head is unmistakable. If peptones are injected directly into the blood of an animal, they act as poisons of a very virulent type, allied to the ptomaines and like bodies, which chemistry has succeeded in extracting from decaying and putrifying meat. It seems that peptones prevent coagulation of the blood when directly placed in contact with it, lower the force of the heart's action, and the blood pressure, produce coma or insensibility, give rise to convulsions, and ultimately cause death. These are, in fact, extraordinary statements, because they refer not to any action which is of unnatural or abnormal kinds, but to the ordinary process of digestion as it is naturally conducted. It is not suggested that all nitrogenous or albuminous bodies give rise equally to poisons by reason of their chemical decomposition. What is certain, is that many of them in the shape of peptones have the power of producing serious effects when they are allowed to pass into the general circulation and to be conveyed throughout the frame. On the one hand, therefore, it would appear that the living body demands these albuminous foods as essential parts of its nutritive store, while on the other hand it is demonstrated that as peptones representing the first stage of their journey they may become virulent poisons. This is apparently another anomaly. The way out of the physiological difficulty, however, lies through the liver.

Our peptones are swept up to the liver through the portal vein, which, by the way, it should be remembered is also bringing to the gland other food products from the latter stages of digestion in the intestines. It behooves us, therefore, to see what part is played by the liver in dealing with the erratic peptones which threaten our physical prosperity and welfare in so marked a degree. A tolerably long physiological experience has shown that the liver is really placed like a police official or sentinel at the gateway of the blood. It has long been matter of scientific knowledge that the gland has the power of dealing effectively with poisons of many kinds which have found access to the body. It thus plays the part of an antiseptic organ or disinfecting establishment rendering innocuous substances otherwise destined very powerfully to affect the welfare of the organism at large. Orfila and others, for instance, showed that mineral poisons, such as Arsenic, Lead, Mercury, and the like, accumulate specially in the liver, and this for the reason that the gland has arrested them from passing into circulation. Later on Schiff, Heyger, and others found that on poisons of a much more powerful nature, to wit, Nicotine, Curarine, Daturine, Morphine, and Strychnine, the liver exercised a distinctive action. There followed naturally on the heel of these discoveries the information that the poisonous bodies produced by the decomposition of albumens, peptones, and the like were also intercepted and rendered chemically inert by the useful and watchful liver. Even the poison of the serpent, subtle as it is, is subjected to the same physiological scrutiny by the gland. Poisons which, like snake virus, act at once when introduced directly into the circulation, were known to be harmless when swallowed, and this result was explained on the assumption that the digestive secretions chemically decomposed them, and rendered them innocuous by the time they were absorbed. To-day we see once again the liver action coming to the front in this process as in the mere natural action of dealing with the poisonous peptones our food may contain. If it be true, as seems likely, that in the process of digestion and also in certain disorders of that function, poisonous principles are generated in the intestine, absorbed by the blood and carried by the portal vein into the liver, we receive a fresh addition to the long list of debts which, in a physiological sense, we owe to our livers for guarding us against various actions that seem inseparable from the discharge of the ordinary procesess of our lives.

Swept up into the liver by blood of the portal vein, the peptones are ultimately brought into immediate contact with the cells of the gland. It is in this relationship that we are brought to see more clearly the physiological importance of the living population of the organ. For whatever work is executed by the liver is simply the outcome of the combined labors of these wondrous microscopic particles, which, in the truest sense of the word are the real laborers in the bodily vineyard. What the liver does with the peptones, and what is the exact nature of the changes which it effects upon them, is still matter of physiological speculation, but perhaps we shall not be drawing upon pure hypothesis too largely or stating that which is unsupported by fact if we assume that they are chemically altered by the liver cells to fit them for passing into the blood to renew and replenish the fluid, and in turn to repair the tissues of the body. Physiologists incline to the belief that the peptones are remodeled in the liver to form a substance known as globuline, which is a very complex albuminous substance much in request by the red corpuscles of the blood. Not all the peptones, however, are thus filtered off and passed on to the blood, escaping out of the liver by the hepatic vein, which we saw to be the outlet channel of the liver. Possibly the liver makes the peptones pay toll on their way through its territory, and so much of the peptonic material retained by the liver is probably converted into a substance known as glycogen, of the curious history of which more anon. Such is the first, but not the whole duty of the liver. It is the detective policeman at the gate of the exhibition who keeps pickpockets from gaining admission, or rather it entraps the evil-doers, securely jails them, and liberates them only when it has purged them of their power to do evil. Following upon these facts comes another of practical importance to the physician. A bilious attack may be compared, as regards its symptoms, to a case of poisoning. There is headache, nausea, retching, vomiting, and collapse. On the idea that the liver's duty from one cause or another has been neglected, we can see how a bilious seizure may be a case of poisoning by peptones, which, like the unarrested pickpockets, find their way into the circulation, and produce on the brain and other organs their dire effects, only too well known as concomitants of our highly civilized but artificial existence.

The story of the liver leads us to notice a second chapter in the history of the duties it performs. This recital begins with the discovery by Claude Bernard, many years gone by, that the liver was to be regarded as a sugar-making organ. Here follows an interesting discussion (citing experiments) respecting the mode of the sugar production. There is, however, proof that if the liver itself does not contain sugar, it is at least a repository, or storehouse of some substance or other, which can be changed into sugar by adding a ferment to it. This substance, it is clear, is of the nature of starch, readily convertible into sugar (as in our mouths), and on this substance the name "Glycogen," or animal starch, has been bestowed. We know that glycogen is naturally stored up in the cells of the liver, which thus becomes a kind of purveyor of starch to the organism, while this starch is also found as a natural constituent of the muscles of the white corpuscles of the blood of the brain and other tissues of the body. It is perfectly certain where the liver obtains its starchy store, namely, from the starches and sugars it obtains from the food. For if an animal be fed exclusively on flesh, which contains no starch, the amount of sugar found in the liver is much less than that which results from a starchy diet, while on the latter diet much starch is stored up in the gland.

Now, what are the meanings and purports of this second duty of the liver? Let us remember that Claude Bernard found the blood issuing from the hepatic vein to be rich in sugar, while the portal vein carried none. Hence came the conclusion that the liver really formed the sugar it gave forth to the blood, that it converted its glycogen or starch into sugar, that this sugar, paid out to the blood by the hepatic vein, was the source of animal heat. Suppose this sugar was not so used up in the lungs? Bernard maintained that it passed into the blood, was carried to the kidneys, and gave rise to the disease known as diabetes. These views have been hotly contested. Dr. Pavy, for example, maintained that the blood of the hepatic vein, issuing from the liver, was not richer in sugar than the blood elsewhere; and this seems to be a crushing fact, annihilating Bernard's theory. Again, the heat of our bodies is not produced in our lungs, but in our muscles, so that this part of Bernard's theory has certainly to be surrendered. The truth probably lies here, as elsewhere, in the middle way. It is true the liver has a very marked sugar-producing power, making sugar out of the starch it stores; and it is also true that sugar, which is soluble, is a form of nourishment or pabulum easily adapted for diffusion in the blood, while starch is not. Again, Bernard probably laid too much stress on what he observed to occur after death in the animal liver, and gave too little heed to the possibly different actions which might, and probably do, occur in the liver during life. Dr. Pavy holds that the sugar formation was a *post-mortem* process, and that the real destination of the glycogen of the liver is not to form sugar, but to convert it into fat, and that this fat goes to aid in the production of bile. When sugar is produced by the liver, this is regarded accordingly, not as the work of a healthy organization, but as the result of disease.

Whether or not the liver is a true fat-forming organ is a point open to dispute. Dr. Pavy, as we have seen, maintains that fat formation is part of the work of the gland. The liver cells certainly contain fat globules, while in sundry states of life, best illustrated perhaps by the fate of the Strasburg goose, the liver in a state of food repletion, combined with inactivity, becomes a mass of fat. Again, no doubt, starches and sugars are fat formers, but it is a difficult matter to conceive how glycogen can be converted into fat, so that on grounds of expediency we may perhaps most safely assume the liver simply stores normally a small quantity, but liable to be excessively increased when a more than adequate amount of fat-forming matters are contained in the food, and when deficiency of muscular exercise is otherwise associated.

We now have left for consideration the third chapter in the liver's story, that relating to its duty as a bile-forming gland. The story is told of a science student, who once on a time, when asked a question regarding bile and its history and uses, replied that bile was formed in the stomach, and was used for cleaning carpets. Doubtless this youth was better instructed in the principles of domestic economy than in the facts of physiology. For though bile is decidedly not found in the stomach, it is often used by housewives, who obtain it from the gall bladder of an ox, for removing grease stains from fabrics of various kinds. The student's answer illustrates the great advantage to be derived from following our knowledge to the ultimate end, for curiously enough, what bile does in the way of dissolving grease stains in a carpet, it also does in the way of dissolving the fats of our food in the intestine. Whatever bile may be, and we have already seen it partake of the nature of a waste product, it certainly assists materially in the digestion of fats. So also it aids the absorption by the wall of the intestine of our digested foods (especially fats), while it may be attributed antiseptic properties, in that it serves to retard injurious decomposition of the food. That it has an influence in stimulating intestinal movements, and thus expediting the digestive process is also an ascertained fact. From the experiments of Schiff it is now well ascertained that the liver is perpetually excreting—that is separating bile from the blood—it is also absorbing bile from that fluid, it is always making new bile by means of its cells from blood as the raw material which it is giving forth to the intestine for digestive purposes the old bile which it has absorbed from the blood brought to it by the portal vein. Bile making and bile elaboration are the additional duties which these living workmen (the cells) perform and sustain. It is from the blood of the portal vein, that great inlet of the liver, that bile is formed, and our previous studies have shown us how intimately associated with the clumps of liver cells are the ramifications of that vessel. That the changes which result in the formation of the bile are of complex description is a fact which need not be emphasized. The protoplasm or living matter of the cells of the liver here as elsewhere, is the seat of a chemistry which practically defies explanation. Very intricate in its own composition is bile itself. It is probably the most complex of our bodily secretions. Containing a large percentage of water, it has solids consisting of cholesterin, pigment fats, and minerals, among which common salt is conspicuous, along with Phosphates of Lime, Soda and Magnesia, Carbonate of Soda, Oxide of Iron, and even traces of Silica itself, of Manganese and Copper. Certain curious compounds of Soda are characteristic of this fluid. It is matter of certainty then that only the cells of the liver can form the substances found in the bile, and it is notable that its color is derived from the blood, while the fat it contains also represents the contribution of the liver cells.

Whatever be the exact origin of the bile this much is certain, that its formation represents one of the most complicated of our bodily duties, while its manufacture testifies no less to the extraordinary powers exercised by the living cells, which with an apparent simplicity of structure, are enabled to figure as the agents in chemical processes, defying the efforts of the furthest science perfectly and clearly to explain. The late eccentric, but talented, Mr. Abernethy, lecturing to his class, delivered himself somewhat to the following effect: "Some have considered the stomach a chemical laboratory, others a mechanical contrivance, a third a cook's shop, and so on, but, gentlemen, the stomach is a stomach and nothing else, and its functions cannot be imitated in any way out of the living body," and he might have added that the same definition would apply to all the other organs of our economy. As Dr. Wilson remarks, the living cells with an apparent simplicity of structure, effect chemical processes, defying the furthest science to explain. Although the anatomical conditions in the cells or protoplasm of the different organs, with the exception of the liver, are apparently the same, which view is further sustained by the example of vicarious secretion, under which the kidney excretes bile, and milk is found in various organs other than the breasts, yet assuming this view to be correct, it would appear that the different products in the different organs can depend alone on the different supplies of nervous energy which they respectively receive. This, then, is a fact of vast importance to us, in our dynamic method of cure.

Each cell of the liver, in addition to capillary arteries and veins, which it enjoys in common with the cells of the other organs, has the inlet capillaries from the portal system, the capillary tubes of the bile ducts and the minute fibrillæ of the nerves which determine the manufacture of the product, which the liver is known to eliminate. To recapitulate: each of these microscopic cells is permeated by, No. 1, the capillary artery; No. 2, the capillary vein; No. 3, the portal capillary; No. 4, the bile duct, and No. 5, the nervous fibrillæ. We must then agree with Dr. Wilson, that these living cells perform one of the most extraordinary and complicated of our bodily duties. What then is the lesson which Dr. Wilson's anatomy, pathology, and physiology teach us? First, to avoid crude, inefficient and mischievous practice, and second, to substitute for it one of a diametrically opposite tendency. Let us here cite the pathalogical example adduced by the doctor. A bilious attack may be compared, as regards its symptoms, to a case of poisoning; there is headache, nausea, retching, vomiting, and collapse. On the idea that the liver's duty, from one cause or another, has been neglected, we can see how the bilious seizure may be a case of poisoning by peptones, which, like the unarrested pickpockets, find their way into the circulation, and produce on the brain and in other organs, their dire effects, only too well known as concomitants of our highly civilized but artificial existence. He does not consider himself called on to enlighten us, as to the treatment, which such studies, on which the edifice of rational medicine is reared, should afford. The case in point is one in which rational medicine would be called on to remove the *materies morbi* from the blood and to prevent their recurrence, to cure the liver. In my younger days the first object would have been attempted by venesection. But now our allopathic brethren know that taking a wine-glass full of brandy and water out of a tumbler will not diminish the proportion of brandy in the remainder. The only escape then for these poisonous peptones will be through the portal system and the liver, but as their original cause was the failure of that organ to perform its duty, it must be whipped up to make time. Hence, as certain substances are known to act on it, they are brought into requisition. *Mercury* is known in a general way to exercise an action on it, hence it is prescribed, commonly as Calomel, the ordinary dose being, say, gr. x. Now what happens? This dose is absorbed by the blood and carried to every part of the body, producing its pathogenetic effects, usually no part of the system escaping its baneful influence. Now let us suppose the said Mercury to be really indicated, what should we homœopathists do? Knowing that the liver being in a state homœopathic to Mercury, will be acted on by a dose which will have no effect on the other parts of the body, to which it is not homœopathic, should give it in a dose sufficiently small to act on the liver alone, leaving all parts of the body, to which it is not homœopathic, intact.

When I began the study of medicine, some sixty years ago, I was indentured, as was then the custom, to a surgeon, with whom the bulk of my duty was to compound and dispense prescriptions, a favorite one of which was a blue pill at bedtime and a black draught in the morning. Thinking over the *rationale* of this routine, I said to him one day: "Doctor, what do you give the blue pill for?" He answered, "It is an alterative." "What is that?" said I. "It is a medicine that makes a change of some sort in the system." "It is a good thing, then?" said I. "Yes, of course," said he. "What do you give the black draught for?" He answered, "To work off the blue pill." "But," said I, "if it is a good thing, why do you want to work it off?" He replied: "You go and put up your medicines." And yet my patron was a first-rate surgeon and of high natural ability. He lived to be ninety, and I never knew him to enjoy a well day, having in his youth suffered from climatic diseases contracted in India and South America. He had little faith in medicine. When I returned from Europe a homœopath, he said: "You have missed it." I answered: "You don't believe in your own physic." "No," said he. "I was dying when A, B, and C held a consultation over me. A said one thing, B said another, but C said: 'We know nothing about it. Let him alone, and give him a chance.' They did so, and if they had physicked me I should have died."

One more of my early reminiscences and I have done: We had a cholera patient in collapse. The doctor said to me: "Mr. Fisher, take sixteen ounces of blood from him." I bandaged his arm and punctured the vein, when, contrary to my expectation (for I thought him too far gone to bleed), the blood spurted out black as ink, but as it flowed from the vein he died as if his throat had been cut. This being the height of a fearful cholera epidemic, I never found the time or opportunity to ask my patron what benefit he had expected to derive from the venesection.

The story of the liver, being too long for a homœopathic monthly, I have extracted what is essential for our purpose, let me, however, again recommend its perusal *in extenso*, for the sake of its anatomy, physiology, pathology, and theory.

The *Harper* number costs some thirty cents, and the lay reading in it is well worth the price.

